# Working on eye-level: The Kinzigtal-way to produce value and health gain—some insights into the cooperation of health, nursing and social services

**Published:** 2012-09-04

**Authors:** Helmut Hildebrandt, Werner Witzenrath

**Affiliations:** CEO of OptiMedis AG and of Gesundes Kinzigtal GmbH, Germany; Primary Care Physician (GP) and Founding Partner of Gesundes Kinzigtal, Germany

**Keywords:** value, efficiency gain, outcome orientation, regional health management company, eye-level, health promotion, prevention

## Abstract

Located in Southwest Germany, Gesundes Kinzigtal develops a fully integrated health care over all sectors of care for the population of the region on a shared gain contract with two statutory health insurers which cover nearly half of the population. It is run by a regional health management company in cooperation with a physicians’ network and a health sciences based health care management company.

Having started in 2006 the more effective trans-sector organisation of the local health care system and increased investments in well-designed preventive and health promotion programmes have led to a reduction in morbidity and mortality. The results for one of the insurers show a substantial morbidity adjusted efficiency gain already for the years 2007–2010, rising to more than 16% of total costs. The workshop will share insights into some of the interventions, which were most effective, and discusses the challenges which had to be overcome. A key-word seems to be to produce an “eye-level paradigm of management” between doctors and patients as well as betweeen all the health professions and between management and providers. More information can be found on www.optimedis.de and www.gesundes-kinzigtal.de and on the evaluation (in German and English) www.ekiv.org

Powerpoint presentation available at http://www.integratedcare.org at congresses – San Marino – programme.

